# Clinical Experience With Nasogastric Tube Placement Using a Biologically Transparent Illumination System in Critically Ill Patients

**DOI:** 10.7759/cureus.97545

**Published:** 2025-11-23

**Authors:** Shinichi Ijuin, Haruki Kaneda, Keisuke Ikeda, Taiki Moriyama, Takeshi Nishimura, Shota Kikuta, Akihiko Inoue, Satoshi Ishihara

**Affiliations:** 1 Emergency and Critical Care Medicine, Hyogo Emergency Medical Center, Kobe, JPN

**Keywords:** biologically transparent illumination system, critically ill patients, enteral nutrition, intensive care unit, nasogastric tube

## Abstract

A biologically transparent illumination (BTI) system has been proposed as an adjunctive method for confirming nasogastric tube (NGT) placement by visualizing a light in the stomach. However, its clinical applicability in critically ill patients remains unclear. We conducted a preliminary prospective observational study to evaluate the clinical feasibility and detectability of a BTI system for NGT placement in critically ill patients. Ten critically ill patients underwent NGT placement using a BTI system after admission to the intensive care unit (ICU). The detected BTI light was assessed in the epigastric region, and the NGT tip was subsequently confirmed using X-ray. The BTI light was visible in five patients, while X-ray confirmed stomach placement in all. Notably, in patients with obesity, massive ascites, or a considerable distance between the NGT tip and the skin surface, the BTI system did not detect the light. No complications during the procedure were reported. This early clinical experience indicates that although BTI detectability is limited by patient-specific anatomical factors, the system may serve as a supportive, hypothesis-generating approach for bedside confirmation of NGT placement in critically ill patients. Further studies are needed to identify the patient groups that benefit most from the BTI system.

## Introduction

Nasogastric tubes (NGTs) are commonly used for feeding, draining fluids and air, and administering medications directly to the stomach. Misplacement of NGT in the respiratory tract, esophagus, or against the gastric wall can lead to serious complications, including aspiration pneumonia, tracheal or pulmonary perforation, pneumothorax, pneumomediastinum, and gastrointestinal perforation [1]. While several methods, such as auscultation, capnography, pH testing, gastric aspirate appearance, and ultrasonography, exist to verify NGT placement at the bedside, none are entirely reliable. Consequently, X-ray examination remains the gold standard for confirming the position of the NGT tip [2,3]. However, it also presents some disadvantages, including delayed verification, medical staff burden, and radiation exposure [4].

Biologically transparent illumination (BTI) is a highly bio-permeable red light from a light-emitting diode (LED). BTI emitted from the tip of a catheter within the abdominal digestive tract is visible from outside the body in adults [5]. Additionally, the BTI system has been demonstrated to be safe and effective for use in children [6].

However, the evaluation of the BTI system has not yet been assessed in intensive care unit (ICU) patients with critical conditions, such as a full stomach, massive ascites, or significant edema. Therefore, we conducted a prospective observational study to evaluate the clinical feasibility of BTI-assisted NGT placement in critically ill patients and to identify clinical and anatomical factors influencing the successful detection of BTI light. This study was designed as a pilot investigation to generate preliminary data regarding the potential usefulness and limitations of this novel technique in the ICU setting.

## Materials and methods

Methods

Study Design and Setting

This was a single-center, preliminary, prospective observational study. Patients were brought to our institution based on emergency medical service judgments and then admitted to the ICU. Participants were enrolled from April 1, 2025, to May 31, 2025. Data and clinical features of patients who underwent the BTI system were collected. This study was approved by the ethical committee of Hyogo Emergency Medical Center (approval number: 2024016). Written informed consent for using patients’ data was obtained from their families at the time of admission by the attending physician.

**Figure 1:**
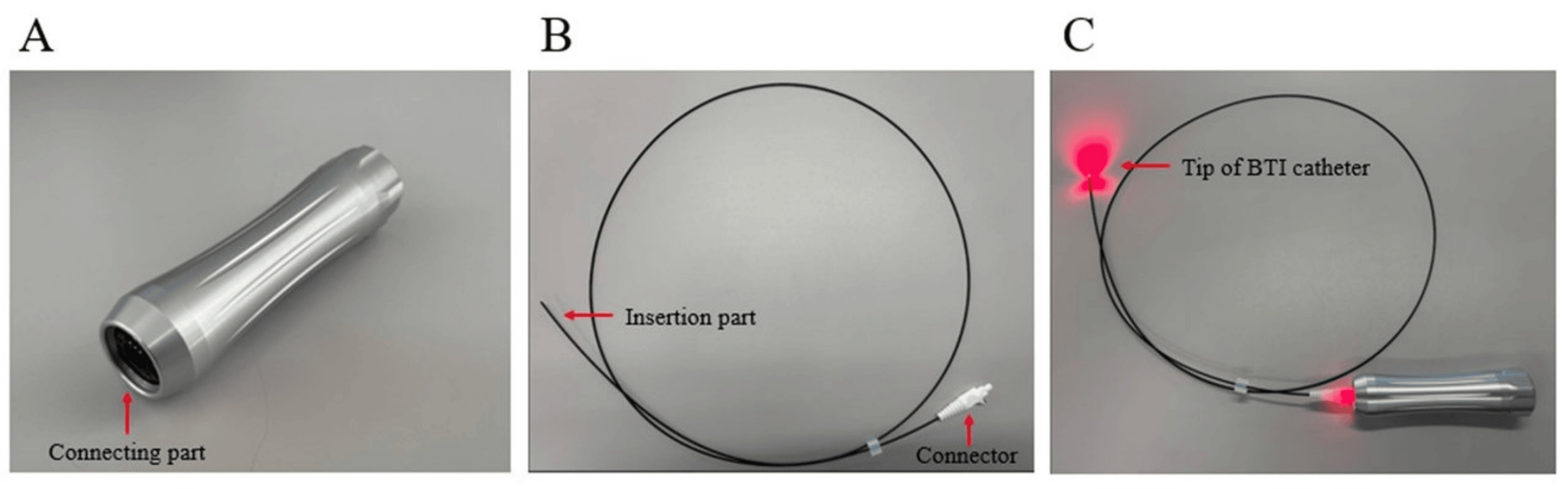
Components of the BTI system (A) BTI light source, (B) BTI catheter, and (C) BTI light is transferred to the tip of the BTI catheter. BTI, biologically transparent illumination Image credits: Authors

BTI system: The BTI device employs a highly bio-permeable red light emitted from an LED light source (Tumguide® LED Light Source: 47B2X10003000001; Otsuka Clinical Solutions, Inc., Okinawa, Japan, distributed by Otsuka Pharmaceutical Factory, Inc., Tokushima, Japan). This light is channeled through a BTI catheter (Tumguide® fiber: 47B2X10003000002; Otsuka Clinical Solutions, Inc., Okinawa, Japan, distributed by Otsuka Pharmaceutical Factory, Inc., Tokushima, Japan), which features plastic optical fibers and has a diameter of 1.0 mm or 1.5 mm. BTI light, with a wavelength of 660 nm, effectively penetrates soft tissues but not hard tissues, such as bone and cartilage. Inserting the BTI catheter into the NGT transfers light to its tip (Figure 1). This emitted light can be visually confirmed from outside the body, allowing precise identification of the NGT tip position within the abdominal digestive tract. Therefore, by using the BTI, the NGT tip position in the stomach can be determined by observing the light in the epigastric and hypochondriac regions [7].

Study Population

This study included 10 consecutive critically ill patients aged 18 years or older, requiring NGT placement in the ICU.

Procedure: The physician determined whether an NGT (New enteral feeding tube (10Fr); Cardinal Health™, Tokyo, Japan) was needed for enteral nutrition. All procedures were performed by a board-certified intensive care specialist, who completed a brief hands-on training session using a bench model provided by the device manufacturer to ensure consistent handling of the BTI system. The assessor was not blinded to the patients’ clinical characteristics. Two types of BTI catheters with different diameters were available during the study period. The selection of catheter type was left to the discretion of the attending physician. Individual ICU rooms were maintained under darkened conditions, with room lights turned off and curtains closed to minimize ambient light and optimize visualization of the illumination signal during the procedures. Following BTI catheter insertion, the NGT was gently advanced from the nostril to the laryngopharynx. After insertion of the NGT to approximately 50 cm-55 cm, the epigastric area was scanned systematically from the midline to both hypochondria for up to 30 seconds. If no light was detected, gentle finger pressure (approximately 1 kgf-2 kgf) was applied over the epigastrium for a few seconds to improve visualization. Once the BTI light was identified, the BTI catheter was carefully removed from the NGT. Then, 10 mL of air was injected, and auscultation was performed to listen for gurgling sounds at the site. Finally, an X-ray confirmed the NGT position, with interpretation by the physician as the reference standard.

Data Collection

We collected the following patient data: age, sex, height, body weight, diagnosis, fiber size used (1.0 mm or 1.5 mm), successful NGT tip placement in the stomach, complications such as nasal or laryngopharyngeal hemorrhage and tracheal loss, as well as the sensitivity and specificity of the BTI system. Additionally, abdominal wall thickness, measured via computed tomography (CT) just below the 10^th^ rib on the midclavicular line, was assessed to evaluate the impact of obesity [5]. CT was also used to measure the shortest distance from the NGT tip to the external body surface. Obesity was defined as body mass index (BMI) >27.5 kg/m^2^ [5]. Continuous variables are presented as median (interquartile range).

## Results

**Figure 2:**
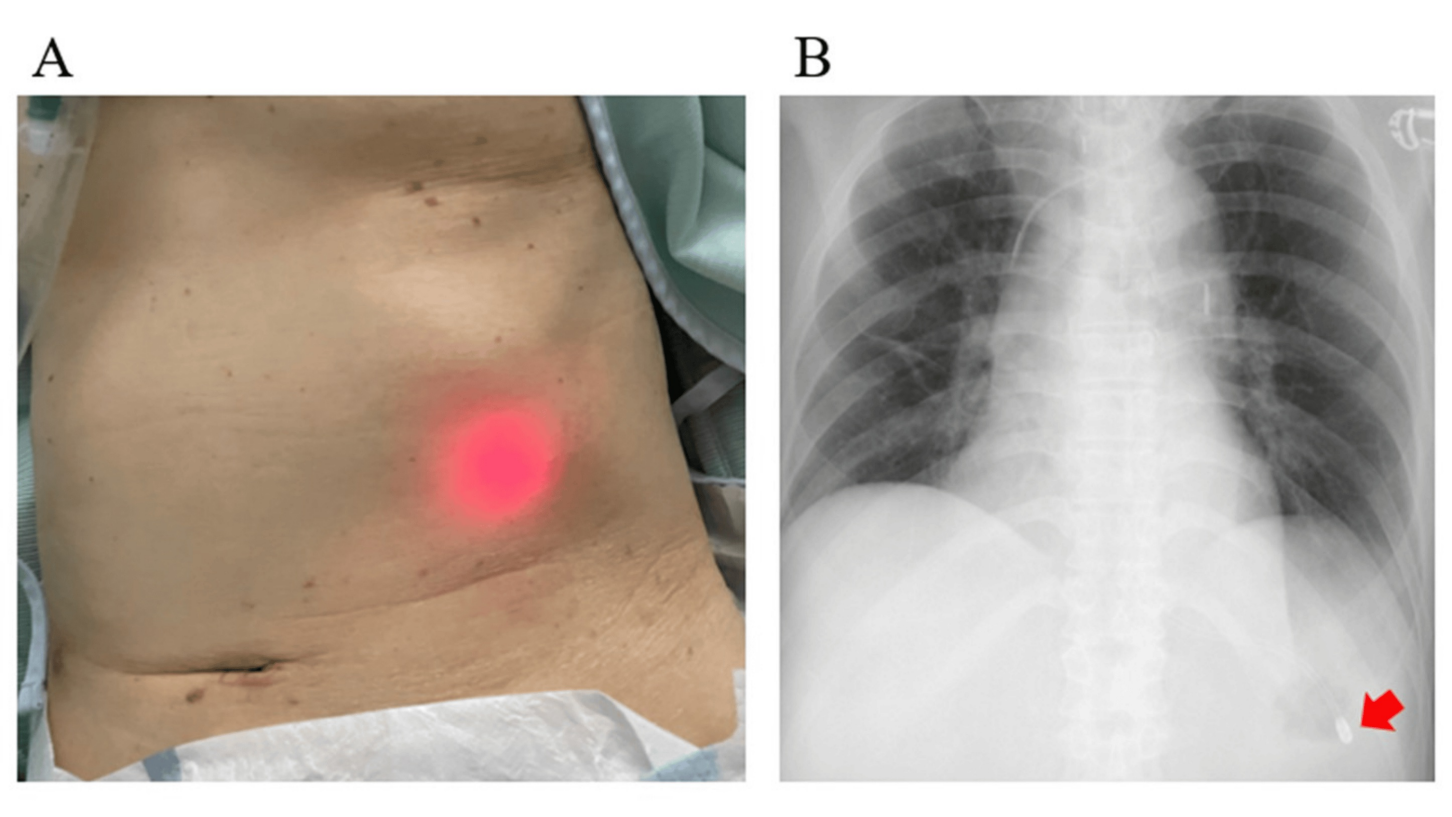
Positive BTI light and X-ray image (case 4) (A) The BTI light was detected in the epigastric area. (B) Corresponding X-ray image. The arrow indicates the NGT tip. BTI, biologically transparent illumination; NGT, nasogastric tube

Table 1 summarizes the patient characteristics. The median age was 73 years, with five patients being male. Diagnoses for admission were as follows: out-of-hospital cardiac arrest (OHCA) (n=3), intracranial hemorrhage (ICH) (n=2), trauma (n=2), sepsis (n=2), and acute pancreatitis (n=1). The median BMI was 24.7 kg/m^2^ (20.3-27.3), including three patients with obesity (cases 6-8). Five patients used the 1.0 mm BTI catheter, while the remainder used the 1.5 mm. Five of the patients were identified as having NGT tip placement in the epigastric area, with BTI light visualization by the BTI system (Figure 2). X-ray confirmed the NGT tip was in the stomach for all patients. The mean abdominal wall thickness was 19.4 mm; it was 17.8 mm (11.6 mm-19.4mm) in patients for whom BTI light was detected and 30.3 mm (19.3 mm-33.5 mm) in patients for whom it was not detected. Among the patients without BTI light detection, only one had a notable distance from the NGT tip to the external surface (case 10). No complications occurred during the procedure.

**Table 1:** Patient characteristics in critically ill patients using the BTI system ^1^Abdominal wall thickness was defined as the measurement taken just below the tenth rib on the midclavicular line by CT. ^2^Distance from the NGT tip to the surface was defined as the shortest distance measured from the NGT tip to the external body surface by CT. ^3^Day 0 was defined as the day of the NGT placement. ^4^BT-light detection was defined as the confirmation of BTI light signal in the epigastric area by a physician. ^5^X-ray detection was defined as the confirmation of the position of the NGT tip in the stomach by X-ray. ^6^No image was defined as the abdominal CT was not performed after the NGT placement. Data are presented as median (interquartile range) for continuous variables. BTI, biologically transparent illumination; BMI, body mass index; NGT, nasogastric tube; ACS, acute coronary syndrome; OHCA, out-of-hospital cardiac arrest; PCAS, post cardiac arrest syndrome; ICH, intracranial hemorrhage; CT, computed tomography

Case	Age	Sex	Diagnosis	BMI (kg/m^2^)	Abdominal wall thickness^1^ (mm)	Distance from the NGT tip to outside^2^ (mm)	CT day^3^	BT catheter size (mm)	BT-light detection^4^	X-ray detection^5^
1	57	M	ACS	24.3	19.4	27.6	2	1.5	Yes	Yes
2	59	M	OHCA - PCAS	21.9	21.1	No image^6^	-	1.5	Yes	Yes
3	77	F	ICH	19.7	11.6	No image	-	1.5	Yes	Yes
4	77	F	Trauma	18.9	10.7	23.7	2	1	Yes	Yes
5	77	F	Sepsis	19.7	17.8	No image	-	1	Yes	Yes
6	60	F	Trauma	27.9	30.3	46.5	1	1	No	Yes
7	70	M	OHCA - PCAS	28.7	33.5	No image	-	1.5	No	Yes
8	76	M	ICH	27.8	33.7	No image	-	1.5	No	Yes
9	79	F	Acute pancreatitis	26.0	19.3	38.8	1	1	No	Yes
10	65	M	Sepsis	23.5	18.5	79.2	1	1	No	Yes
	73	-	-	24.7 (20.3-27.3)	19.4 (17.9-28.0)	-		-	-	-

## Discussion

This preliminary prospective observational study presented an initial clinical experience with the BTI system-assisted NGT placement in critically ill patients. In this small cohort, external visualization of the BTI light was achieved in half of the cases, while radiographic imaging confirmed appropriate gastric placement in all patients. These findings suggest that although the BTI system can be observed under certain anatomical conditions, its detectability appears to be influenced by factors. Therefore, it suggests that the BTI system has limited usefulness in unselected critically ill patients and may only be applicable to specific subgroups without anatomical or environmental limitations. Although the number of cases was limited, the procedure itself was simple and did not result in adverse events. Given its minimally invasive nature, this method may offer supplementary value for confirming intra-gastric NGT tip placement in critically ill patients. To our knowledge, the application of the BTI system has not previously been assessed and reported in critically ill patients. Consequently, this study may be the first document of its application in an intensive care setting.

Traditional X-ray examination has been the gold standard for confirming NGT placement, but it leads to radiation exposure and delays in confirmation. Therefore, new methods have been developed to verify the correct NGT position (e.g., a fiberoptic pH test device, direct vision-guided, and electromagnetic trace-guided tube placement [8-10]). However, each approach presents its challenges, such as expense and the requirement for expert training, which must be addressed before it can be used routinely in clinical settings.

The advantage of the BTI system is its non-invasiveness and rapid results. As previously reported, it provides a quick and safer alternative [5,7]. Its ability to visually confirm BTI light presence externally reduces the necessity for repeated X-ray examinations, thereby mitigating radiation risks. Furthermore, this system allows the NGT placement to be performed without a burden on medical staff, such as radiologists.

**Figure 3:**
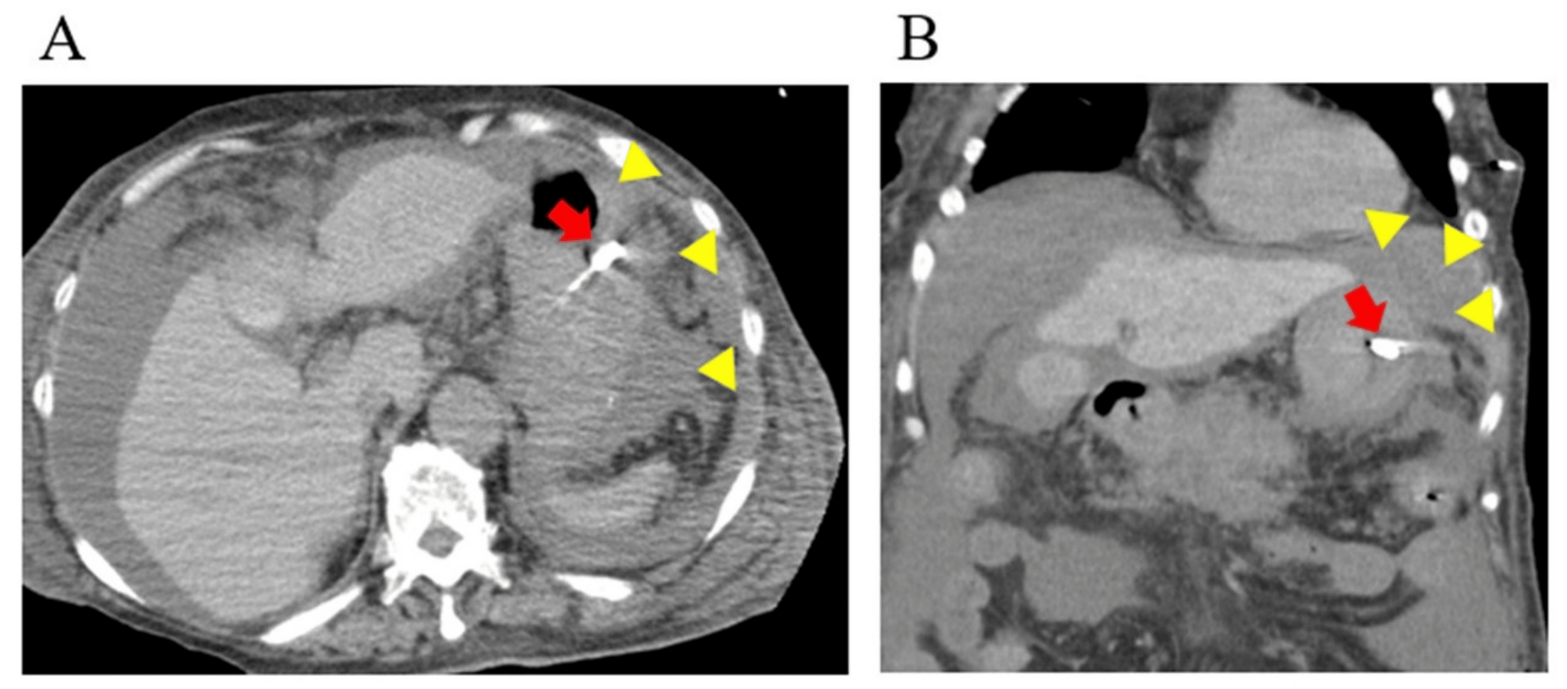
CT image on the day after the NGT placement (case 9) The image shows the NGT tip in the stomach (arrow) and massive ascites (triangle). (A) shows the axial scan, and (B) shows the coronal scan. CT, computed tomography; NGT, nasogastric tube

**Figure 4:**
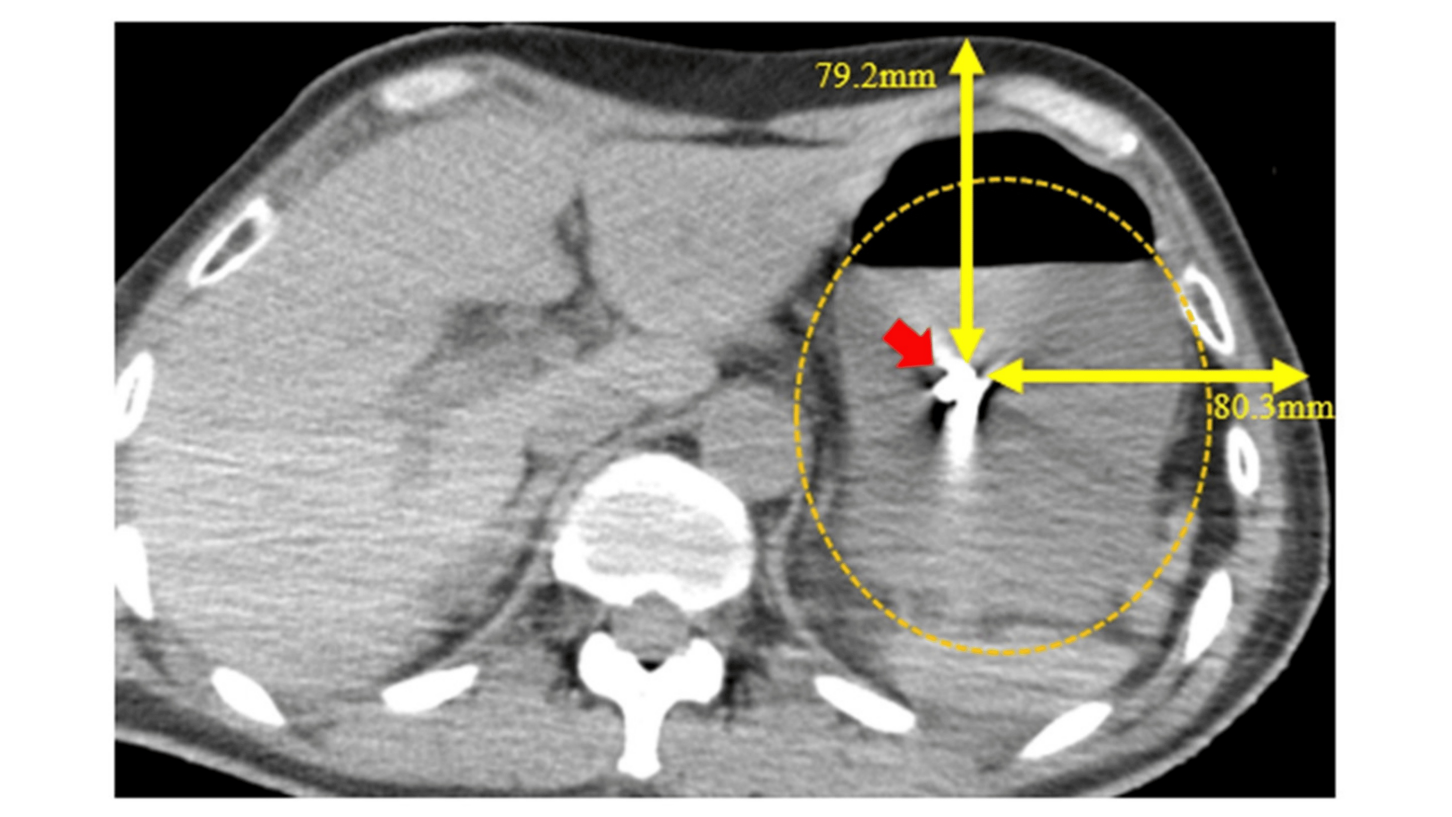
CT image on the day after admission and NGT placement (Case 10) This image shows the NGT tip was away from the abdominal wall (79.2 mm) and buried in the gastric residue. The arrow indicates the NGT tip, the double arrow shows the distance from the NGT tip to the abdominal wall, and the circle marks the gastric residue. CT, computed tomography; NGT, nasogastric tube

However, in this study, the BTI light was not confirmed from outside the body in five patients. Detecting light in the stomach would be particularly challenging for obese patients (cases 6-8) due to their thicker abdominal walls. The light detection may be difficult when abdominal wall thickness exceeds 20 mm, measured by CT [11]. Additionally, another study demonstrated that the distance from the BTI catheter tip to the body surface is greater in patients with obesity than in those without [10]. In case 9, a patient with massive ascites due to acute pancreatitis was not detected by the light (Figure 3). Furthermore, in case 10, a patient with a large gap between the NGT tip and the external surface, along with significant gastric residue, also lacked detectable BT light (Figure 4). Consequently, the BTI light may be too weak to reach outside the body due to the increased distance from the NGT tip and barriers such as ascites or gastric residue.

Previous studies have reported favorable detection rates of BTI light in selected patients, such as elective surgical patients with adequate fasting periods and pediatric patients with thin abdominal walls [5-7]. In contrast, the present study indicates that the BTI system may not be suitable for critically ill patients, such as those with obesity, residual gastric volume, or substantial ascites. Therefore, this investigation should be interpreted as a preliminary, hypothesis-generating clinical experience rather than a confirmatory evaluation of BTI system performance. The results demonstrate that while BTI-assisted NGT placement is technically feasible and safe, its detectability is strongly affected by anatomical and physiological conditions common in the ICU population. Consequently, the BTI system may be considered a situationally useful adjunctive tool rather than a universally reliable verification method.

This study has several limitations. First, it was a single-center study. Second, the sample size was very small, with no control group. Therefore, this investigation should be interpreted as a pilot feasibility study intended to generate preliminary clinical experience and hypotheses rather than to evaluate efficacy. Third, the physician assessing the BTI light visibility was not blinded to the patients’ clinical characteristics, such as body habitus or the presence of ascites, which could have introduced expectation bias in the subjective assessment of light visibility. Fourth, although the assessments for the BTI system were performed in darkened ICU rooms (lights off and curtains closed), minor variations in ambient light may still have influenced the perception of the BTI light. Fifth, two types of BTI catheters with different diameters were used, as both were available in our institution during the study period, and the selection of a catheter was determined by the attending physician. Differences in catheter diameter may have affected the light permeability and visibility of the BTI light, and this factor should be considered when interpreting the results. Sixth, only one type of NGT was inserted under intubation/sedation and in the supine position. Since various types of NGTs, made from different materials, serve different purposes in clinical practice, our results may not be generalizable when using a different NGT type without sedation, intubation, or in a non-supine position. Seventh, the timing of CT acquisition relative to NGT placement varied among cases. In several patients, CT was performed on the day after NGT insertion or later; therefore, the anatomical measurements obtained from CT may not precisely reflect the conditions at the time of evaluation of the BTI system. Consequently, the identified anatomical characteristics are associations rather than proven causal determinants. Finally, the patients’ characteristics were heterogeneous, so it’s unclear whether these findings can be applied broadly to all critically ill patients.

## Conclusions

This preliminary observational study describes an early clinical experience with BTI-assisted NGT placement in critically ill patients. While the technique was feasible and free from procedural complications, the limited detectability of BTI light highlights its dependence on patient anatomy and clinical condition. These findings should be viewed as hypothesis-generating observations that warrant validation through larger, controlled studies designed to determine the true clinical utility and optimal indications of BTI technology in critical care.
